# CD38 Expression by Myeloma Cells and Its Role in the Context of Bone Marrow Microenvironment: Modulation by Therapeutic Agents

**DOI:** 10.3390/cells8121632

**Published:** 2019-12-13

**Authors:** Federica Costa, Benedetta Dalla Palma, Nicola Giuliani

**Affiliations:** 1 Department of Medicine and Surgery, University of Parma, 43126 Parma, Italy; federica.costa2@studenti.unipr.it (F.C.); benedetta.dallapalma@gmail.com (B.D.P.); 2 Hematology, “Azienda Ospedaliero-Universitaria di Parma”, 43126 Parma, Italy

**Keywords:** CD38, multiple myeloma, monoclonal antibodies

## Abstract

In the last decades CD38 has emerged as an attractive target for multiple myeloma (MM). CD38 is a novel multifunctional glycoprotein that acts as a receptor, adhesion molecule interacting with CD31 and as an ectoenzyme. As an ectoenzyme, CD38 functions as a metabolic sensor catalyzing the extracellular conversion of NAD+ to the immunosuppressive factor adenosine (ADO). Other ectoenzymes, CD73 and CD203a, together with CD38, are also involved in the alternative axis of extracellular production of ADO, bypassing the canonical pathway mediated by CD39. CD38 is ubiquitously expressed in the bone marrow microenvironment; however, only MM cells display a very high surface density, which lead to the development of several anti-CD38 monoclonal antibodies (mAbs). The efficacy of anti-CD38 mAbs depends from the presence of CD38 on the surface of MM and immune-microenvironment cells. Interestingly, it has been reported that several drugs like lenalidomide, panobinostat, the all-trans retinoic acid and the DNA methyltransferase inhibitors may increase the expression of CD38. Hence, the possibility to modulate CD38 by increasing its expression on MM cells is the pre-requisite to potentiate the clinical efficacy of the anti-CD38 mAbs and to design clinical trials with the combination of anti-CD38 mAbs and these drugs.

## 1. Introduction

Multiple myeloma (MM) cells are characterized by tight relationship with the bone marrow (BM) microenvironment which supports their growth, survival, and induces drug resistance. MM cells overexpress several surface adhesion molecules that are involved in the relationship with the micro-environmental cells like BM stromal cells (BMSCs), osteoblasts (OBs), and endothelial cells [1]. Among the adhesion molecules, CD38 is highly expressed by MM cells. CD38 has become one of the main targets for monoclonal antibody (mAb) therapy in MM patients [2]. Daratumumab (DARA) is the first anti- CD38 mAb approved for the treatment of relapsed and refractory MM based on the results of two phase I/II trials [3]. Other anti-CD38 mAb were also clinically developed as isatuximab and MOR202 [4]. The combination of anti-CD38 mAbs with main anti-MM drugs as proteasome inhibitors (PIs) and immunomodulatory drugs (IMiDs) demonstrated a high clinical efficacy in randomized phase III trials [5] leading to new therapeutic paradigms in relapsed/refractory MM patients. However, a considerable amount of MM patients does not respond or are refractory to the treatment with anti-CD38 mAbs either as single agents or in combination with PIs or IMiDs [6,7,8].

The clinical efficacy of anti-CD38 mAbs seems to be related, at least in part, to the intensity of CD38 expression by MM cells and other cells of the immune-microenvironment. The possibility to modulate CD38 through an increase of its expression by MM cells is the pre-requisite to potentiate the efficacy of anti-CD38 mAbs. Different pharmacological agents have demonstrated the capacity to increase the expression of CD38 by MM and BM microenvironment cells. This review summarizes the main experimental evidences on this topic giving the rational for drug combinations with anti-CD38 mAbs in the treatment of MM.

## 2. CD38 Expression by MM Cells and BM Microenvironment

MM is a hematological cancer characterized by the accumulation and proliferation of malignant plasma cells (PCs) in the BM [9]. The close interaction between PCs and BM microenvironment cells creates a permissive niche for tumor survival and disease progression, characterized by osteolytic bone disease and immune-suppression [10]. Both soluble factors and cell-to-cell contact mechanisms are involved in this cross-talk. Among the surface molecules, which allow the adhesion to the microenvironment, MM cells highly express CD38 [11], which made it an attractive therapeutic target for mAbs [12,13]. Several studies demonstrated that only PCs strongly express CD38 antigens in BM, and that no PCs are detectable in either CD38^neg^ cell fraction or fraction of cells weakly expressing CD38 antigens (CD38^low^) [14,15]. However, activated B cell, T cells and NK cells up-regulate CD38 surface expression to levels similar to that found on PCs [16].

CD38 is a 45-kDa type II transmembrane glycoprotein, which plays a dual role as a receptor and ectoenzyme [17]. It is expressed on normal cell subsets, such as T cells, NK cells, B cells, and dendritic cells [18]. It is involved in T cell activation and proliferation, B cell differentiation, and neutrophils and monocytes chemotaxis, [17,19,20]. In addition, CD38 interacts with the non-substrate ligand CD31, which is constitutively expressed by endothelial cells [21]. Interestingly, a co-expression of CD38 and CD31 was also demonstrated in MM cells but not on PC leukemia [22]. Accordingly, we have recently reported that extra-medullary MM cells can also lose the expression of CD38 [23].

As ectoenzyme, CD38 acts like a metabolic sensor which catalyzes the extracellular conversion of NAD+ to regulators of calcium signaling, such as adenosine (ADO), according to pH status [24]. Other ectoenzymes, CD73 and CD203a, together with CD38, are involved in the alternative axis of extracellular production of the immunosuppressive factor adenosine ADO, bypassing the canonical pathway mediated by CD39 [25].

ADO levels are significantly higher in the BM plasma of MM patients than asymptomatic monoclonal gammopathies as Monoclonal Gammopathy of Uncertain Significance (MGUS) and smoldering MM (SMM), suggesting that ADO is produced in the MM niche by an ectoenzymatic CD38 network [26]. Recently, we have also investigated the expression and function of ectoenzymes on microvesicles (MVs) isolated from BM plasma samples of MM patients. Our results show that the percentage of MVs expressing high levels of ectoenzymes was higher when derived from MM patients compared to MGUS and SMM. Consistently, BM CD138^+^ PCs from MM patients expressed high levels of all ectoenzymes [27]. The MVs immunophenotype of MM patients indicated a high expression level of CD38, CD39, CD73, and CD203a ectoenzymes as shared by CD138^+^ PCs. Finally, we demonstrated that the ATP, NAD^+^, ADPR, and AMP to ADO catabolism was higher in MVs from MM patients than in those from controls. This indicates that the ectoenzymes expressed by MVs isolated from BM samples of MM patients were functionally active and involved in the higher ADO production as compared to MGUS and SMM [27].

It is known that the interactions between MM PCs and other cells of the BM niche, such as osteoclasts (OCs), OBs, and BMSCs, induce the production of ADO, which promotes tumor survival and immune escape [28,29,30]. Several studies also reported that ADO concentrations in the BM MM niche correlates with disease progression and may be an useful prognostic marker related to ISS staging together with others [26]. In addition, it has been interestingly shown that the fully humanized anti-CD38 mAb, DARA, is able to modulate CD38 enzymatic activity in vitro, thus reducing ADO levels and reverting its immunosuppressive effect [28].

Analysis of CD38 and the other ectoenzyme distribution within MM bone niche revealed that only PCs express high levels of CD38 [31]. However, some studies showed that CD38 expression intensity is highly heterogeneous on MM cells, and its expression does not differ from newly diagnosed and relapsed/refractory MM patients [32]. MM PCs also express other ectoenzymes like CD39 and CD73, although their levels differ from patient to patient [31].

The expression profile of BMSCs and OBs show that both cell types are CD38^−^/CD39^−^ while expressing CD73 and CD203a [26,30,31]. Indeed, CD38 decreases during OB differentiation with a concomitant increase of CD203a [30].

By using murine and rabbit models, studies from Sun L. et al. [33,34] described an involvement of CD38 in the remodeling of the adult skeleton [33,34]. Specifically, a reduced bone mineral density was detected in CD38^−/−^ mice, along with a strong ability of the hematopoietic stem cells to differentiate into highly resorptive OCs [34]. CD38 was also detectable on rabbit OC plasma membrane where it exerts an ADP ribosyl cyclase activity [34]. Bone resorption was inhibited after the treatment with an anti-CD38 agonist antibody, further confirming CD38 involvement in skeleton remodeling [33]. These studies also supported the hypothesis of CD38 as a metabolic sensor able to couple OC bone resorption activity with its Ca^2+^ signaling pathway [35].

However, few data are currently available on CD38 expression on human monocytes and OCs in MM. A recent study from Costa F. et al. [31] shows that CD38 is expressed on the surface of early OC progenitors but it is lost during in vitro differentiation toward an osteoclastogenic phenotype [31]. Moreover, in vitro experiments demonstrated that the use of the fully humanized anti-CD38 mAb, DARA, inhibits OC formation and activity, confirming the involvement of CD38 in bone remodeling, even in MM patients [31].

Besides their major function in bone remodeling, OCs also display immunosuppressive properties on T cells [36] which in turn induce osteoclastogenesis [37], thus establishing a feedback loop mechanism. An G. et al. [38] recently demonstrated that anti-CD38 mAb, SAR650984, enhances the cytotoxic effect of T cells on OCs in vitro [38]. On the other hand, the presence of SAR650984 in T cells-OCs co-cultures restores T cell proliferation, reverting the OC immune-suppressive effects [38]. Overall, these data suggested that targeting CD38 may affect MM-induce bone disease, by restoring T cell function or inhibiting early OC formation.

Lastly, Krejcik J. et al. [16] suggested that CD38 expression may define a subset of T regulatory cells (Tregs) with enhanced immune suppressive effect [16]. Studies on MM revealed that Tregs expresses CD38 at higher level compared to T constitutive cells (Tcons). Moreover, the percentage of CD38^high^ cells is higher in MM patients versus healthy donors [16,39]. In vitro co-culture also showed that MM cells significantly induces Tcons conversion toward a Treg phenotype, which is characterized by an increased expression of CD38, CD25, and FoxP3 compared with natural Tregs [39].

Together all these data indicate a pleiotropic role of CD38 in MM bone niche, supporting the use of therapeutic strategies targeting this molecule to inhibit MM cell growth, survival, and to revert immune-suppression and bone disease in MM patients.

## 3. CD38 Modulation by Anti-CD38 Monoclonal Antibodies in MM Cells

Several anti-CD38 mAbs have been developed and are under clinical development: Two fully human Abs (MOR202, TAK-079) and one chimeric (isatuximab); while DARA is the yet approved for the treatment of MM as single agent and in combination with standards of care in relapsed/refractory MM patients [40].

Anti-CD38 mAbs show different mechanisms of action: Fc-dependent immune effector mechanisms, direct effects, and immunomodulatory effects. The first mechanism includes complement-dependent cytotoxicity (CDC), antibody-dependent cell-mediated cytotoxicity (ADCC), antibody-dependent cellular phagocytosis (ADCP), and apoptosis upon secondary cross-linking [40]. The direct effect is mediated by caspase-dependent apoptotic pathway, as well as the lysosomal cell death pathway [41]. Finally, it has been recently reported that DARA treatment induces elimination of CD38-positive immune suppressor cells, such as Tregs, regulatory B cells, and myeloid-derived suppressor cells [16,42], together with activated NK cells [43], suggesting DARA ability to target cells with lower levels of CD38.

Despite the in vitro effects results and the well-established clinical efficacy of the anti-CD38Abs, some open issues may occur. Specifically, a study from Nijhof IS et al. [8] reported that CD38 expression is reduced in both BM and PB CD138^+^ cells, together with non-tumor immune cells (NK, T cells, B cells, and monocytes) following the first DARA infusion and it increases again following DARA discontinuation [8]. Different mechanisms have been proposed to explain this event. One is that the interaction of DARA with its target induces a polar aggregation, known as “capping”, of CD38 molecules, followed by exocytosis as MVs [28] or endocytosis of DARA-CD38 complex [44]. Another mechanism involves the degradation of the antigen–antibody complex or the rapid elimination of myeloma cells expressing high levels of CD38, as demonstrated by the fact that MM CD38 levels were only reduced in the presence of complement or effector cells. More recently, it has been suggested a mechanism called “trogocytosis”, where fragments of the plasma membrane carrying antigen–antibody complexes, together with other parts of cell membrane (CD49d, CD56, CD138, CD54, and CD44) are transferred by monocytes and granulocytes, in the absence of evident phagocytosis of tumor cells [44]. This process seems to be involved the reduction of CD38 not only on MM cells but also on NK cells. Interestingly, these in vitro studies were then confirmed in vivo in patients treated with DARA, both in the presence or absence of lenalidomide [44].

Since drug-response was maintained in many patients despite the decreased CD38 expression by MM cells [44], events other than those just described may be responsible for treatment failure. Nonetheless, repeated treatment with DARA is possible and efficacious [43].

Therapeutic combination with agents that increase CD38 expression, such as panobinostat an–histone deacetylase inhibitor [45] and trans retinoic acid [32], can overcome these limitations as demonstrated by the increased of anti-CD38 mAb-mediated ADCC in vitro [32,45]. More details are reported in the following section.

Recently, Moreno L et al. [46] described that in vitro treatment of several human myeloma cell lines with another anti-CD38 mAb, isatuximab, induces CD38 internalization without its release from MM cell surface [46]. The authors found that isatuximab-mediated ADCC, ADCP, and CDC are triggered only in the presence of a certain number of surface CD38 molecules [46].

All together these data thus suggest that not all patients could benefit from the same anti-CD38 mAbs and combination with other therapeutic agents should be investigated.

## 4. Drug-Mediated Modulation of CD38 Expression by MM Cells

The expression of CD38 by MM cells is potentially modulated by different agents used in the treatment of MM or for other hematological malignancies.

Firstly, authors investigated the role of all-trans retinoic acid (ATRA) on the expression of CD38 by MM cells. Low doses of ATRA were able to increase up to 4.4 fold CD38 expression on MM cell lines. Accordingly, ATRA significantly increased CD38 expression in all the BM mononuclear cells of MM patients. Moreover, in a humanized mouse model ATRA enhances DARA mediated ADCC and CDC against MM cells either in vitro or in vivo [32]. Similarly, we recently observed that the inhibitory effect of DARA on OC formation is significantly enhanced by ATRA treatment. This treatment increased CD38 expression by monocytes and early OC progenitors [31].

There are different mechanisms involved in the modulation of CD38 by ATRA. Previous studies have demonstrated that the retinoic acid receptor has an important role in the induction of CD38 by ATRA [47]; indeed, the CD38 gene contains a retinoic acid-responsive element. Non-classical retinoic acid signaling is also involved in CD38 up-regulation, independently from the conventional retinoic acid receptor pathway [48]. Among these mechanisms, signaling mediated by protein kinase Cδ [48] and phosphatidylinositol 3-kinase [49] have been described.

IMiDs are agents able to act on both MM and microenvironment cells by targeting several adhesion molecules [50]. Specifically, it was reported that the integrin-signaling pathway is significantly modulated by lenalidomide and pomalidomide in MM cell lines. In particular, CD38 was up regulated after IMiDs treatment whereas ITGA8 and ICAM2 (CD102) were both down-regulated [51]. More recently, Fedele P. et al. [52] reported that lenalidomide treatment increases CD38 expression in several human myeloma cell lines, through Ikaros and Aiolos degradation. Thereafter, the authors tested the hypothesis that the additive effect observed by the combination of anti-CD38 mAbs and IMiDs in vitro could be due to the upregulation of CD38 by IMiDs [52]. They found that the additive effect of the treatment combination was directly correlated with the increased CD38 surface expression on MM cells [52]. However, in the presence of lenalidomide it was not observed any additional effect on DARA direct cytotoxicity [52]. On the other hand, lenalidomide potentiated DARA-induced ADCC against MM cells by directly stimulating NK cells without modifying CD38 expression on these cells. Overall these evidences indicate that the clinical synergistic effect of the combination of lenalidomide and DARA is likely due either to the increased CD38 expression on MM cells or to the stimulation of NK activity [52].

Panobinostat is a pan-histone deacetylase inhibitor (HDACi) approved for the treatment of MM relapsed patients in combination with bortezomib [53]. Recent data indicate that panobinostat is able to up-regulate the expression of CD38 by primary PCs either in newly diagnosed MM or in relapsed MM patients [45]. Interestingly, this effect seems to be specific for MM cells and does not occur in lymphoma cells [45]. Consistently with the effect of panobinostat on the expression of CD38 by MM cells, it has been demonstrated that the cytotoxic effect of DARA was increased by the treatment of panobinostat [45]. A significant increase of ADCC was observed against panobinostat pre-treated cells compared to untreated myeloma cells in all BM samples obtained from MM patients [45]. Interestingly, panobinostat treatment did not affect CD38 expression on other cell types as T cells [45].

The mechanism by which panobinostat increases CD38 expression may involve the interaction between Ikaros and the nucleosome remodeling deacetylase (NuRD) complex, known to regulate several transcriptional events involved in oncogenesis and cancer progression [54]. NuRD contains at least two subunits with an ATP-dependent chromatin remodeling, together with HDAC1/2 subunits, which catalyze protein deacetylation [54]. Co-immunoprecipitation experiments demonstrated that Ikaros interacts with HDAC1/2 in MM cells, by sharing similar binding patterns in the CD38 locus [52]. It is thus conceivable that Ikaros-induced CD38 repression is mediated by its interaction with HDAC1/2. These results were further supported by the synergistic upregulation of CD38 after combination treatment with lenalidomide and low doses of HDAC- inhibitor panobinostat [52].

Together with the pan- HDACi, panobinostat, the effect of the class I HDAC and HDAC6 inhibitors were investigated. The class I HDAC-specific inhibitor is able to up-regulate CD38 expression by MM cells and to increase the effect of interferon (IFN)-α and ATRA, in several MM cell lines tested. On the other hand, HDAC6 inhibitor attenuated the CD38 expression induced by IFN-α and ATRA. Similarly, these authors found that panobinostat attenuates IFN-α and ATRA up-regulated expression of CD38 [55]. It is known that the upstream sequence of CD38 gene contains an interferon regulatory factor 1 (IRF1)-binding site [56,57] suggesting the role of STAT-1-IRF1 pathway activation in the up-regulation of CD38 by IFN-α. A dual opposite effect was thus observed regarding the different type of HDAC inhibitors [55]. Indeed, IFN-α and ATRA up-regulation of CD38 expression was attenuated by HDAC6 inhibitor, ACY-1215, but not by the class I HDAC1 inhibitor, MS-275 [55]. These in vitro evidences suggest that HDAC class I inhibitors could be used in combination with ATRA to potentiate the effect of anti-CD38 mAbs.

CD38 was identified as a differential methylating region in MM patients with a negative correlation between DNA methylation and gene expression. Moreover, analysis of different independent MM datasets showed an inverse relationship between normal PCs and malignant MM cells in terms of CD38 methylation and gene expression status [58]. Accordingly, it has been recently demonstrated by flow-cytometry that DNA methyltrasnferase inhibitors (DNMTi), as azacytidine (AZA) and decitabine (DEC), increase CD38 expression on a panel of human MM cell lines, without affecting cell viability [58]. Furthermore, the combination of AZA or DEC with ATRA, significantly increased surface CD38 expression as compared with the treatment with the single drugs [58]. Next, by using immortalized NK transgenic cell line, the authors showed a significant increase in DARA-mediated-ADCC against MM cells pre-treated with DNMTi as compared to not-treated cells [58]. The mechanism behind the AZA-mediated upregulation of CD38 expression by MM cells involved TNF-α signaling as demonstrated by the abrogation of DNMTi effect in the presence of neutralizing TNF-α antibody [58].

## 5. Clinical Results of Anti-CD38 mAbs in Combination with Agents Able to Upregulate CD38 Expression in MM Patients

Overall the evidences indicate that different pharmacological agents can modulate the expression of CD38 with a possible translational effect. Published data suggests that the use of IMiDs, ATRA, and panobinostat may increase the clinical efficacy of anti-CD38 as DARA, isatuximab and MOR202. The combination of DARA with IMIDs have been extensively tested in several studies on MM patients at different stages of disease. The outstanding efficacy of the combination of DARA with lenalidomide have led to a rapid approval of the regimen in relapsed/refractory MM patients, demonstrating an overall response rate (ORR) of 92.5% and a progression free survival (PFS) at 12 months of 83.2% [7]. The same regimen has been tried in the first-line setting, showing a complete response (CR) or better in 47.6% of patients, with a 70.6% of patients without disease progression at 30 months [59]. DARA has also been tested in combination with pomalidomide, showing promising results in a phase Ib trial, even in heavily pretreated patients and in patients exhibiting high-risk cytogenetic features (ORR: 60% in the whole population, 59% in high-risk patients, 55% in patients with >3 previous lines of therapy; median PFS: 8.8 months) [60]. Accordingly, it was reported the significant clinical effect of the combination anti-CD38 antibody isatuximab and lenalidomide. A phase Ib trial reported an ORR of 56% with a median PFS of 8.5 months, with no safety concerns [61]. The phase Ib trial of isatuximab in combination with pomalidomide showed even more promising results, with an ORR of 62% and a median PFS of 17.6 months in patients heavily pretreated [62]. The phase III randomized trial is currently ongoing. However, to our knowledge, none of these studies have evaluated the expression in vivo of CD38 on MM cells.

Based on the results of pre-clinical studies, a phase I/II clinical trial of DARA in combination with ATRA has been designed, even if no results have been published yet. Conversely, there is no evidence of possible clinical use of the combination of anti-CD38 mAbs and panobinostat. Results of main clinical trial of anti-CD38 antibodies in combination with drugs that potentially modulated the expression of CD38 by MM cells are reported in Table 1.

## 6. Conclusions

CD38 is a suitable target for immunotherapy in MM patients due to its expression profile in the BM microenvironment. MM cells expressed CD38 at high levels. On the other hand, among the cells of the BM microenvironment it has been demonstrated that NK, T cells, and monocyte express CD38 with different levels of expression. Growing evidence indicate that the efficacy of anti-CD38 mAbs is related, at least in part, to the CD38 intensity of expression by MM cells and those of the immune-microenvironment. The possibility to modulate CD38 increasing its expression by MM cells is the pre-requisite to potentiate the efficacy of anti-CD38 mAbs. Moreover, it has been shown that anti-CD38 mAbs may modulate the CD38 expression on the surface of MM cells by its internalization or capping.

Different pharmacological agents have demonstrated the capacity to increase the expression of CD38 by MM cells and their BM microenvironment. Particularly different experimental data indicate that ATRA is able to increase the expression of CD38. Among the anti-MM drugs, it has been shown that the HDAC inhibitor panobinostat increased CD38 expression by MM cells. The same effect has been found with lenalidomide and pomalidomide. More recently, it has been reported that DNMTi as AZA or DEC also increase CD38 expression by MM cells [58].

**Figure 1:**
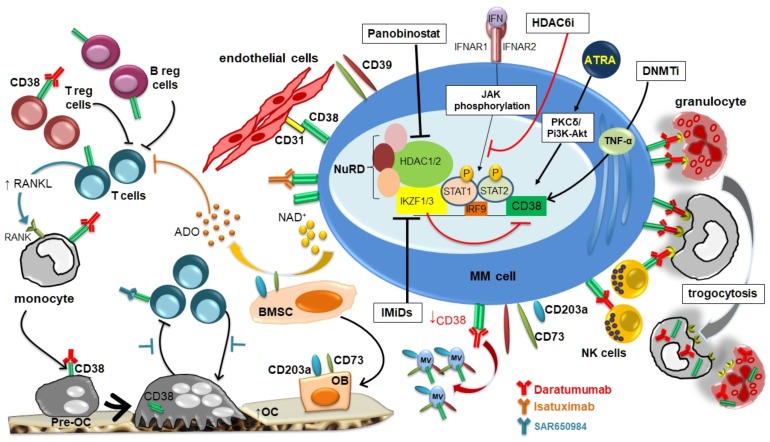
CD38 expression in multiple myeloma (MM) microenvironment and its modulation by different agents.

Figure 1 summarizes the main mechanisms involved in the modulation of CD38 expression in MM cells and in the BM microenvironment by different molecules with a possible therapeutic impact.

These observations provide the rational to design clinical trials using anti-CD38 mAbs such as DARA and isatuximab in combination with IMiDs, HDACi, and DNMTi. Clinical trial showed that the combination of DARA with IMiDs is highly clinical efficient to induce a profound response in relapsed/refractory MM patients.

## Figures and Tables

**Table 1:** Clinical trial of combination of anti-CD38 mAbs with drugs able to modulate CD38 expression.

Drug Combination	Trial Name (NCT number) [Ref.]	Phase	Number of Patients	Primary Outcome	Results Reported	AEs (Grade 3–4)
DARA/ Lenalidomide	54767414MMY3008 (NCT02252172) [59]	III	737	PFS	PFS 70.6% at 30 mos	Neutropenia 50%, lymphopenia 15%
DARA/ Lenalidomide	Pollux Study (NCT02076009) [7]	III	569	PFS	ORR 92.9% *vs* 76.4% Median PFS *nr vs* 17.5 months (HR 0.44)	Neutropenia (54%), anemia (15.5%), pneumonia (12%)
Isatuximab/ Lenalidomide	A Phase 1b Study of SAR650984 (Anti-CD38 mAb) in Combination with Len and Dex for the Treatment of RRMM (NCT01749969) [61]	I	57	MTD of the combination	ORR 56% Median PFS 8.5 months	Neutropenia (60%), lymphopenia (58%)
DARA/Pomalidomide	54767414MMY1001 (NCT01998971) [60]	Ib	103	MTD of the combination	ORR 60% Median PFS 8.8 months	Neutropenia (77%), anemia (28%), thrombocytopenia (19%)
Isatuximab/ Pomalidomide	TCD14079 (NCT02283775) [62]	Ib	45	MTD of the combination	ORR 62% Median PFS 17.6 months	(AEs all grade) Fatigue (62%), upper respiratory tract infection (42%)
DARA/ ATRA	A Phase 1 and Phase 2 Study of DARA in Combination with ATRA in RRMM (NCT02751255)	I/II	60	1) MTD 2) ORR 3) RDL	No result posted	No result posted

Abbreviations: DARA, Daratumumab; ATRA, All Trans-Retinoic Acid; RRMM, Relapsed/Refractory Multiple Myeloma; MTD, Maximum Tolerated Dose; ORR, Overall Response Rate; RDL, Recommended phase 2 dose level; PFS, Progression Free Survival; HR, Hazard Ratio; CBR, Clinical Benefit Rate; AEs, Adverse events.
